# Functional and Clinical Relevance of the Crosstalk between the Glymphatic System and the Lymphatic System

**DOI:** 10.2174/011570159X359861250224051857

**Published:** 2025-06-03

**Authors:** Marsel Khabibov, Airat Garifullin, Amina Sadreeva, Manuel Felipe Fernandez, Marat Kashaev, Iuliia Topchu, Leonid Kharin, Yanis Boumber

**Affiliations:** 1 Department of Radiology, Dmitry Rogachev National Research Center of Pediatric Hematology, Oncology and Immunology, Moscow, 127994, Russia;; 2 Department of Radiation Oncology, Republican Clinical Oncological Dispensary, Ufa, 450054, Russia;; 3 Department of Internal Medicine, I.M. Sechenov First Moscow State Medical University, Moscow, 119992, Russia;; 4 Department of Medicine, Feinberg School of Medicine, Northwestern University, Chicago, IL 60611, USA;; 5 Department of General Surgery, Bashkir State Medical University, Ufa, 450000, Russia;; 6 Robert H. Lurie Comprehensive Cancer Center, Department of Medicine, Division of Hematology/Oncology, Feinberg School of Medicine, Northwestern University, Chicago, IL 60611, USA;; 7 Molecular Therapeutics Program, Fox Chase Cancer Center, Philadelphia, PA 19111, USA;; 8 O’Neil Comprehensive Cancer Center, Department of Medicine, Division of Hematology/Oncology, Heersink School of Medicine, University of Alabama in Birmingham, Birmingham, AL, 35233, England;; 9 Institute of Fundamental Medicine and Biology, Kazan (Volga Region) Federal University, Kazan, 420012, Russia

**Keywords:** Glymphatic system, lymph nodes, aquaporin-4 protein (AQP-4), perivascular spaces, blood-brain barrier, therapeutics

## Abstract

In this review, we describe the concept of the glymphatic system as a glial-dependent clearance pathway in the brain. The hypothesis of the glymphatic system function suggests that dural lymphatic vessels absorb the cerebrospinal fluid and brain interstitial fluid *via* the glymphatic system and transport fluid into deep cervical lymph nodes. We present the accumulated data of various studies confirming the possible interconnection among the brain interstitial fluid, cerebrospinal fluid, and the glymphatic system. Anatomical features are discussed here together with a possible variety of glymphatic system functions, including the removal of waste products, transport of substances, and immune function. The glymphatic system is hypothesized to be involved in pathogenesis of many diseases, including Alzheimer's disease, stroke, and Parkinson’s disease. We also discuss the role of the glymphatic system in pathophysiology and the complications of brain tumors. Meningeal lymphatics is thoroughly analyzed as well. Finally, we propose new treatment approaches to brain tumors, Parkinson’s disease, and stroke using cervical lymph nodes and backward fluid flow in the meningeal lymphatic vessels.

## 1 INTRODUCTION

The central nervous system (CNS) lacks conventional lymphatic vessels [1]. Recently, the roles of meningeal lymphatics, located in the dura mater outside the CNS, and the glymphatic system have become topics of ongoing debate [2, 3]. Despite a disproportionately high level of energy consumption [4], it has remained unclear for a long time as to how metabolic products are excreted from the CNS. The concept of the glymphatic system (GS) was introduced in 2012 as a brain-wide pathway that plays a pivotal role in clearing the brain waste products [2]. According to this concept, fluid from the subarachnoid space first enters the brain parenchyma along the arterial perivascular spaces (PVSs) and mixes with the brain interstitial fluid (ISF). It is essential to distinguish between the para- and perivascular spaces. When pial arteries penetrate the brain parenchyma, they transform into penetrating arterioles, which are surrounded by a perivascular space known as the Virchow-Robin space. Additionally, there exists a space within the arterial tunica media, located between the intermediate layers of the basement membrane of arterial smooth muscle cells. This structure, referred to as the perivascular space, also serves as a conduit for fluid flow [5]. However, in the context of the glymphatic system, we focus exclusively on perivascular spaces here.

**Fig. (1):**
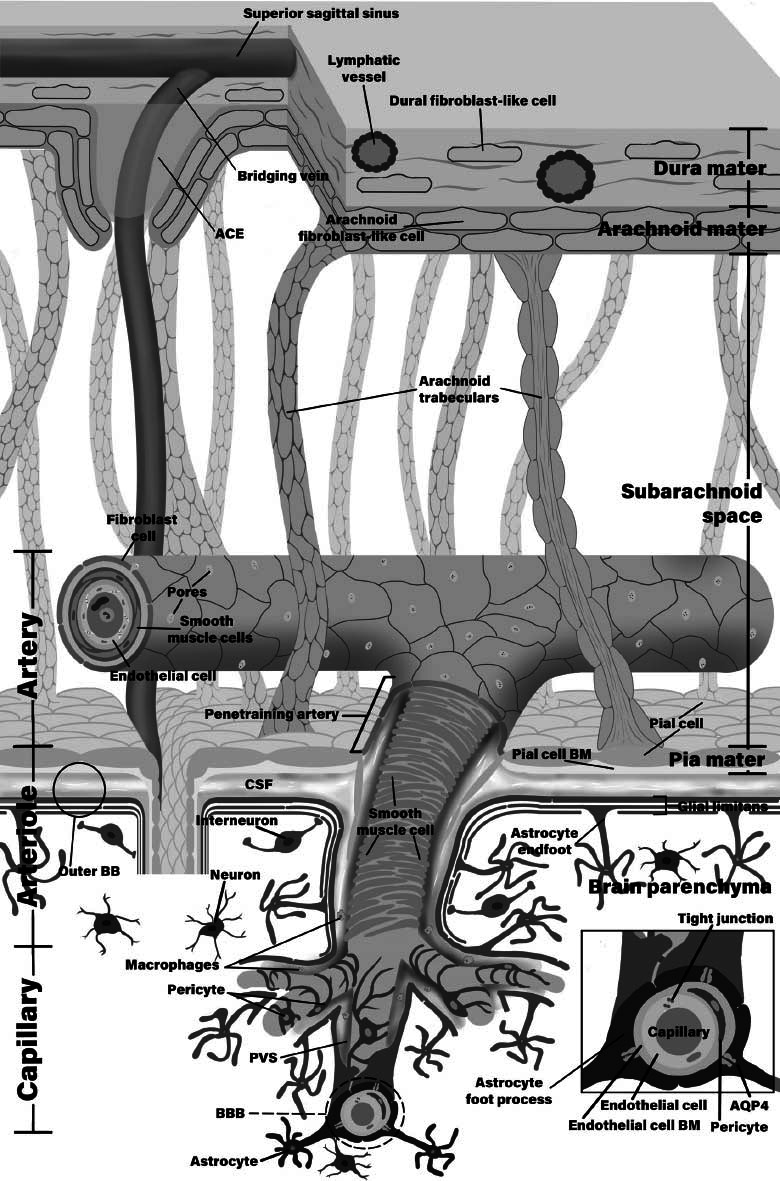
Structural components of the glymphatic system. CSF is produced at the choroid plexus, then it is distributed across the subarachnoid space. From the subarachnoid space, the fluid gets transported through the PVSs of the penetrating arteries to the AQP-4 channels of the astrocyte foot processes. AQP-4 carries the fluid to the brain interstitial fluid, bypassing the BBB. Dural lymphatic vessels channel the fluid to the cervical lymph nodes. **Abbreviations**: ACE - arachnoid cuffs exit, BB - brain barrier, BBB - blood-brain barrier, PVS - perivascular space, AQP-4 - aquaporin-4.

The interstitial fluid then exits the parenchyma along the venous PVSs and is removed from the brain (Fig. **1**). A similar concept was initially proposed in 1985 by Rennels *et al*., who postulated that waste is removed *via* a convection pathway of rapid cerebrospinal fluid (CSF) microcirculation to the brain with perivascular influx around arteries and efflux along veins [6].

Meningeal lymphatic vessels were distinctly described in the 18^th^ century by Paolo Mascagni in his work “Vasorum Lymphaticorum Corporis Humani Historia et Ichnographia” (History and Graphical Representation of the Lymphatic Vessels in the Human Body) [7]. Perivascular spaces (also known as Virchow-Robin spaces, named after the German pathologist Rudolf Virchow (1821-1902) and French anatomist Charles Philippe Robin (1821-1885), that were described by them in 1851 and 1859, respectively) are fluid-filled structures that surround the walls of arteries, arterioles, veins, and venules as they enter the brain parenchyma coming from the subarachnoid space [8, 9]. Interestingly, Virchow and Robin disagreed on whether these spaces directly communicated with the subarachnoid space.

There was an incomplete explanation with respect to the spaces as it was mathematically demonstrated that simple diffusion cannot provide a sufficient level of fluid exchange between CSF and ISF in brain tissue to account for the metabolic demands of the CNS. In addition to simple diffusion, advective fluid transport has also been reported to take place [10]. The concept of the GS (as an active transport system) functions as an explanation for how these processes take place in the CNS. The GS in the brain functions as an analogue of the lymphatic system, and indeed, many similarities have been observed between the two systems (Table **1**) [11-21].

However, differentiating between the meningeal lymphatics (along with the lymphatic vessel) and the glymphatic system is crucial due to their distinct anatomical substrates, despite having somewhat similar functions.

In recent years, several excellent critical reviews have been published on the concept of the glymphatic system [22, 23].

## 2 ANATOMICAL FEATURES OF THE GLYMPHATIC SYSTEM AND THE ROLE OF THE BLOOD-BRAIN BARRIER IN ITS COMPOSITION

The brain is the most complex organ in the human body. It contains roughly 100 billion neurons [24] and consumes 15-20% of the total body energy despite only representing 2% of the body’s weight [25]. It involves a system of brain barriers, including the blood-brain barrier (BBB) and the blood-CSF barrier. There is also an outer brain barrier that is localized between the brain and subarachnoid CSF, with an initial radial glial endfeet layer covered with a pial surface layer, with tight junctions being its basis [26, 27]. Brain interstitial fluid is isolated from the hydro-dynamic effect of the systemic circulation. The systolic force of the heart cannot exert its effect on the interstitial fluid compartment. One could conclude that there is a hydrodynamic shortage required for interstitial flow in the brain tissue. However, water influx *via* the astrocyte aquaporin-4 (AQP-4) water channel into the peri-capillary PVSs and fenestrated capillaries compensates for this shortage and creates a condition nearly identical to systemic circulation with appropriate interstitial fluid motion. Interstitial flow in the periarterial PVSs is hypothesized to be involved in the β-amyloid clearance, whereas interstitial flow in the perivenous PVSs provides ventricular CSF required for normal CSF circulation [28].

BBB is formed by several structures: endothelial cell junctions, vascular wall basement membrane, pericytes, and astrocytic endfeet [29, 30]. The fluid-filled space that contains many different cells, including astrocytes and leukocytes [31] between the vascular basement membrane and the astrocytic endfeet processes layer (commonly named glia limitans) is called PVSs [32]. Electron microscopic 3D reconstruction of perivascular endfeet showed that they almost entirely covered the surface of blood vessels excluding the microglia extending to the vascular basement membrane [33]. PVSs have been reported to form an integral part of the neurovascular unit of the BBB [30] (Fig. **1**).

Astrocytes regulate the brain blood circulation by producing different products that contribute to vessel dilation (*e.g*., prostaglandin E2) or constriction (*e.g*., 20-HETE) [34], thus contributing to glymphatic flow (as it is regulated by the arterial pulsation) [35]. Using photolysis, it was demonstrated that astrocytes regulate cerebral blood flow through the production of prostaglandins that dilate vessels. This activity is inhibited by indomethacin and cyclooxygenase-1 inhibitor [36]. Another study showed astrocytic endfeet to actively close gaps in blood vessel walls created by laser ablation. The authors proposed that astrocyte endfeet play a pivotal role in sustaining CNS in the physiological condition through active maintenance of blood vessels [37].

Another important function of the astrocytes in the brain is water transport *via* the AQP-4 receptor [38]. The basement membrane located under the epithelial and endothelial cells is a particular type of extracellular matrix that provides structural support and cell signaling [39]. The basement membrane is 20-200 nm thick [40] and mainly consists of four major glycoprotein families: laminins, collagen IV isoforms, nidogens, and heparan sulfate proteoglycans [41].

Endothelial cells' tight junctions almost completely limit the extent of passive diffusion of molecules into the brain and lead to a very high trans-endothelial electrical resistance [42]. To allow the shuttle of nutrients and metabolites across the brain, endothelial cells have a special carrier-mediated system [43]. Attempts have been made to employ these carriers to mediate the delivery of different therapeutics to cross the BBB, but these have mostly failed to demonstrate clinical utility due to the limited capacity of such transport systems [44].

Among other components of BBB, pericytes have been shown to regulate BBB activity *in vivo* in rodent models, since pericyte deficiency leads to the increased permeability of BBB. Pericytes also regulate the polarization of astrocytes’ endfeet [45]. In another study, a decreased number of pericytes led to increased BBB permeability [46].

Pericytes regulate the cerebral blood flow by contracting or relaxing depending on neuronal activity [47, 48]. They possess different contractile proteins, including alpha-smooth muscle actin, tropomyosin, and myosin. However, it seems that contractile function is only performed at the arteriolar end of the capillary bed due to the higher expression of alpha-smooth muscle actin [49, 50].

It appears that BBB transport and the GS serve the same function in clearing interstitial solutes, including amyloid beta (Aβ). Accumulation of Aβ in the brain tissues is observed in Alzheimer’s disease. Aβ is transported across the local BBB by efflux transporters, such as LRP1 and P-gp. However, Aβ needs to be cleared through the ISF flow in the GS. It was shown that impaired Aβ clearance is associated with a decline in glymphatic function [51, 52], suggesting a role of the GS in the clearance of Aβ. It is also crucial to assess various aspects of the BBB concerning the clearance of Aβ. One significant protein linked to the development of Alzheimer's disease is the low-density lipoprotein receptor-related protein-1 (LRP1), whose dysfunction may contribute to the disease. Research has shown LRP1 to not only be involved in the uptake of Aβ, but also play a role in its degradation in the brain and its removal from the body. In their study, Storck and colleagues utilized transgenic mouse models that allowed for the conditional deletion of LRP1, specifically in the brain's endothelial cells. The absence of LRP1 resulted in increased levels of soluble Aβ in the brain, which in turn worsened deficits in spatial learning and memory [53]. Another protein that may contribute to this process is the ABC transporter P-glycoprotein (ABCB1/P-gp). The phosphatidylinositol binding clathrin assembly protein (PICALM) serves as a connector between these two proteins. PICALM facilitates the transport of the abluminal Aβ-LRP1 complex to Rab11-positive sorting endosomes, where ABCB1/P-gp is located. This interaction promotes the efflux of Aβ by ABCB1/P-gp on the luminal side of the endothelial cells [54].

An article published in 2023 described a potentially fourth meningeal layer, the subarachnoid lymphatic-like membrane (SLYM). It could compartmentalize the subarachnoid space into an outer superficial compartment and an inner deep compartment lining the brain in both humans and mice [55]. However, in the subsequent commentary on this article, it was argued that the two-photon imaging conducted in Prox1-EGFP+ reporter mice did not reveal a previously unknown cell layer within the subarachnoid space. Instead, it indicated the presence of the already well-known neurothelial cell layer, located beneath the inner membrane of the dura mater and above the arachnoid barrier cell membrane [56]. It is also important to note that fixatives are hyperosmotic and could lead to the shrinkage and dislocation of brain membranes [57].

## 3 AQP-4 WATER CHANNEL AND ITS IMPLICATIONS

AQP-4 water channel is thought to play a key role in the transport of fluid from CSF (subarachnoid space) to the ISF and a pivotal role in the function of the GS. It is very important to consider its role when talking about the backward fluid flow.

AQP-4 is one of 13 similar mammalian aquaporins, several of which have a wide tissue distribution and a complex range of activities [58]. AQP family members are transmembrane proteins that are characterized by the six transmembrane α-helices and intracellular carboxyl (C) and amino (N) terminal domains [59]. AQP-4 is the primary water channel in the brain [60] and it is mainly expressed in the perivascular astrocyte foot processes [61]. AQP1 and AQP9, by contrast, are mainly detected in pathologic states [62]. It has been proposed that AQP9 may aid in the clearance of lactate and glycerol from the extracellular space in the setting of cerebral ischemia [63]. AQP1 expression in human astrocytes is generally restricted to conditions, such as multiple sclerosis, Alzheimer’s disease, Parkinson’s disease, and amyotrophic lateral sclerosis [64-66]. Nine isomers of AQP-4 are identified in different species. However, only M1, M23, ex, and Δ4 isoforms have been found in human astrocytes [67]. M1, M23, and ex isoforms form orthogonal arrays [68, 69]. AQPs conduct passive water transport because the direction of flow always follows the main osmotic gradient [70].

AQP-4 has a range of functions in the CNS, including the regulation of extracellular space volume, potassium buffering, cerebrospinal fluid circulation, interstitial fluid resorption, waste clearance, neuroinflammation, osmotic sensing, cell migration, and Ca^2+^ signaling. AQP-4 is also necessary for retinal, inner ear, and olfactory systems function [71].

In addition to these well-established functions, new roles of AQP-4 have been proposed. Nedergaard and colleagues suggested that the glymphatic clearance mechanism, in which solute transports from cerebrospinal to interstitial fluid, involves AQP-4-dependent convection in the brain parenchyma [2]. However, Smith *et al*. found no difference in CSF tracer inflow into the brain parenchyma of AQP-4 knockout (KO) mice compared to wild-type controls [72]. Mestre *et al*. refuted the data of this latter study. They conducted a re-evaluation of the impact of Aqp4 deletion on perivascular glymphatic exchange using data from five different laboratories. The results of this study consistently showed that Aqp4 deletion impairs perivascular glymphatic flow compared to wild-type mice. They used four independently created Aqp4 KO lines, including the line utilized by Smith *et al*., as well as the α-syntrophin KO line, which lacks AQP-4 perivascular localization despite normal expression levels. All these four lines injected in mice showed the transfer of intra-cisternally injected tracers from the CSF into the parenchyma to be highly impaired [73].

It is also important to note that AQP-4 was initially thought to be a pure water channel protein [74]. However, according to the concept of the GS, AQP-4 plays a role in the clearance of all waste products [2]. AQP-4 could also play a role in osmosensing [75].

Igarashi *et al*. studied AQP-4 inhibitor-TGN-020 in a mouse model of focal cerebral ischemia. This work suggested that AQP-4 inhibition might be used pharmacologically to reduce brain edema caused by localized ischemia [76]. Popescu *et al*. discovered the use of AQP-4 inhibitor to have a positive health effect. TGN-020 clearly lowered infarct volume and brain hemisphere volume in TGN-020-treated mice compared to untreated animals. The impact was apparent in the contralateral hemisphere, but at a decreased level [77].

AQP-4 is a target of specific antibodies in a spectrum of inflammatory CNS disorders, especially neuromyelitis optica, again suggesting its relevance in health and disease [78].

The initial trigger of autoimmune response remains an important question. The perivascular system and its interconnection with neck lymph nodes may be an additional link to the pathogenesis of aquaporin-4 autoimmunity. Moreover, some lesions due to AQP-4-IgG antibody exhibit marked inflammation and astrocytic AQP-4 loss [79], which can lead to GS deterioration.

AQP-4 is the main water channel in the brain and is proposed to play a critical role in glymphatic transport. This is confirmed by several studies in which the AQP-4 loss has led to the impairment of tracer transport from CSF to the brain interstitium. As antibodies to AQP-4 are associated with inflammatory CNS disorders, AQP-4 inhibitors could be a viable therapeutic option in the future.

## 4 CEREBROSPINAL FLUID

The choroid plexus, which forms the blood-CSF barrier, is mostly responsible for the production of cerebrospinal fluid. It exists as a fibrous network of tissue and vasculature in the ventricles. Choroid plexus epithelial cells secrete numerous neuropeptides, growth factors, and cytokines. Receptors involved in transcytosis and polypeptide clearance are also widely expressed in the choroid plexus [80]. Ependymal cells line the ventricles of the brain and the central canal of the spinal cord. They also regulate the production and circulation of cerebrospinal fluid (CSF), as well as contribute to brain metabolism and waste clearance [81].

CSF is absorbed and drained by arachnoid granulations *via* the lymphatic pathway that incorporates fluid escape by means of cranial and spinal nerve sheaths, which may account for up to 50% of CSF [82]. Tracers injected into the CSF in one study entered and exited the brain *via* separate periarterial basement membrane pathways. Fluorescent fixable amyloid was injected into the cisterna magna CSF. The pons and caudoputamen achieved the greatest depths of tracer penetration into the brain. This research study suggested that CSF may be used as an appropriate route for the delivery of therapies for neurological diseases, such as cerebral amyloid angiopathy [83, 84]. It is also important to remember that many different structures are responsible for the clearance of CSF. Research indicates that arachnoid granulations are not directly connected to the venous sinuses. Instead, fluid from these granulations freely diffuses into the perisinus and diploic areas. Arachnoid granulations may function as reservoirs for cerebrospinal fluid (CSF) and act as immune hubs at the interfaces of the meninges [85]. Additional studies have revealed significant changes over a lifetime in the quantity, size, and distribution of arachnoid granulations within the superior sagittal sinus and adjacent cranial bones. However, many individuals with a completely normal CSF system lack arachnoid granulations in the dural sinuses, raising questions about their role in CSF physiology [86]. Meningeal lymphatic vessels, especially those at the base of the skull, also play a role in the drainage of CSF. They have lymphatic valves and capillaries located adjacent to the subarachnoid space in mice. Additionally, it has been shown that their structure is impaired with aging [3]. Bridging veins create gaps in the arachnoid barrier, leading to the formation of arachnoid cuff exit points (ACE points). These structures enable fluid and molecular exchange between the subarachnoid space and the dura mater, facilitating cerebrospinal fluid drainage and the restricted movement of molecules from the dura to the subarachnoid space. Additionally, in conditions, like experimental autoimmune encephalomyelitis, ACE points allow immune cells to access the subarachnoid space directly from the dura mater, promoting cellular trafficking [87]. In the context of aging and Alzheimer's disease, compromised drainage of the meningeal lymphatic system contributes to the accumulation of toxic misfolded proteins in the CNS. This impairment may be associated with the persistent elevation of meningeal interferon-gamma [88].

While PVSs play a huge role in the GS, it is especially difficult to characterize these spaces as they practically disappear in the postmortem brain [89]. When an animal dies, the periarterial spaces are lost, according to two-photon imaging. CSF tracers are displaced into the surrounding smooth muscle layer and basal lamina as the perivascular space contracts and eventually disappears [90].

Animal studies have demonstrated a connection between the subarachnoid space and deep cervical lymph nodes through the cribriform plate [91, 92], one around the internal jugular vein and another through dural lymphatic vessels located in the skull base and parasagittal areas [3, 14].

The study by Smyth *et al*. offered significant insights into the anatomy of the arachnoid barrier and its implications for fluid dynamics in the brain. The finding that the arachnoid barrier surrounding bridging veins is incomplete suggests that CSF can traverse from the subarachnoid space into the dura mater. This has implications for gaining an understanding of both the glymphatic and meningeal lymphatic systems [87].

**Fig. (2):**
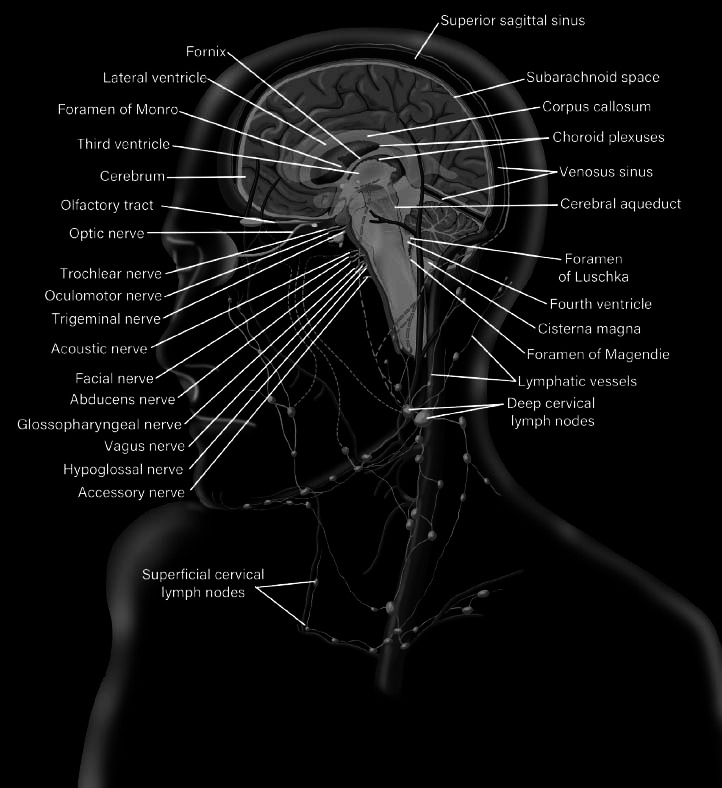
Cerebrospinal fluid circulation system (with main anatomical structures) and its connection to the cervical lymph nodes. The fluid gets absorbed at the meninges and is transported by the meningeal lymphatic vessels (the dotted green line) to the deep cervical lymph nodes. The GS connects subarachnoid space (shown as light green space) to the brain parenchyma with its interstitial fluid.

Compelling experimental data demonstrated connections between the lymphatic system of CNS and deep cervical lymph nodes in humans [93]. Utilizing cadaver models, researchers visualized lymphatic endothelial cells with an anti-podoplanin antibody, and immune cells were labeled using an anti-CD45 antibody. They were able to confirm that lymphatic vessels from the intracranial space run through the dura and appear to connect with the deep cervical lymphatic network (Fig. **2**). Interestingly, lymphatic vessels are located within the dura around the jugular foramen, but not around the cranial nerves or around the venous part of the foramen.

In the human and rodent brains, the PVSs communicate with the subarachnoid space, as evidenced by multiple studies showing that tracer uptake is visible in PVSs following *in vivo* administration of tracers into CSF (vide infra) [94].

One study highlighted an interesting point regarding the structure of the eye. The aqueous humor is collected *via* Schlemm’s canal, which despite morphological and functional similarities with the lymphatic vascular system, is not developmentally identical to lymphatic vessels, as it requires angiopoietin expression [95]. Therefore, if a small structure like the eye requires a specialized draining system, larger and more complex structures like the brain also require similar draining pathways.

Studies by the groups of Roy Weller and Roxana Carare, using fluorescent tracers and laser confocal microscopy, have proposed ISF and solutes to drain from the mouse brain along the basement membranes of capillaries and arteries. This could be a route for ISF drainage from the brain to the cervical lymph nodes. It is suggested that this perivascular lymphatic drainage pathway is obstructed by amyloid-beta in CAA and Alzheimer's disease [96].

CSF outflow outside the discussion of the glymphatic system has also been reviewed elsewhere [97].

## 5 FUNCTIONS OF THE GLYMPHATIC SYSTEM

In this section, we discuss the function of the GS in systemic brain waste transport, CSF, and intracranial pressure (ICP) regulation, and its potential role in metabolic and neurotransmitter homeostasis.

### 5.1 Fluid Outflow

While the importance of the GS in the outflow of interstitial fluid is still under discussion [98], it plays an important role in interstitial flow dynamics, which is critical for metabolism. As aforementioned, owing to the tight junctions of the capillary endothelium of BBB, the interstitial fluid system of the brain cannot benefit from the hydrodynamic force of the systolic pulse of the heart. The main role of astrocyte AQP-4 water channel is to provide appropriate water influx into the pericapillary space and maintain necessary hydrodynamic conditions for interstitial flow [28]. Therefore, the AQP-4 receptor is a key structure that provides adequate interstitial flow in the brain.

### 5.2 Lipid Transport and Signaling

As the central nervous system lacks conventional lymphatic vessels, GS may serve as an analog for lipophilic molecules’ transportation. It has been shown that the brain has a distinct perivascular compartment for lipid transport and glial signaling. Violation of perivascular transport causes increased intracellular lipid accumulation and abnormal astrocyte calcium signaling [21].

Additionally, almost all glutamate and potentially most of the γ-aminobutyric acid is uptaken by the glial endfeet in the brain [91]. It has been proposed that the GS plays a key role in the glutamate/glutamine and GABA glutamine cycle [99, 100].

### 5.3 Elimination of Metabolic Waste Products

GS is the source for clearing toxins and metabolic waste products from the brain, thereby being an essential mechanism in restoring the functions of a healthy brain [2]. Interstitial space expands during the sleep. All the processes of GS function during sleep are marked by a doubling of the CSF clearance rate along with a 60% increase in the interstitial space in the brain during non-wake states as compared to wake states [101].

The mechanisms underlying the accumulation of Aβ in the Alzheimer’s disease brain are still incompletely understood. Aging-associated decrease of perivascular recirculation of CSF/ISF has been proposed as a driver of accumulation of Aβ in the brain parenchyma [2, 72, 51]. It has been demonstrated that levels of Aβ in brain interstitial fluid increase with time spent awake and decrease with time spent asleep [102]; limited sleep hours and greater amyloid-β accumulation are associated with Alzheimer's disease [103]. There are suggestions that GS also provides clearance of alpha-synuclein, underlying the neuropathology of PD, as well as Lewy body disease and multiple system atrophy [104]. Interestingly, increased efficiency of the GS has not only been demonstrated in natural sleep, but also during other sleep-like states. GS transport changes similar to slow-wave sleep were also found during ketamine/xylazine anesthesia [101]. Moreover, blockade of adrenergic signaling without sleep or anesthesia expanded interstitial fluid volume, accelerated GS transport, and was associated with slow-wave electrocorticography activity [101]. It is also proposed that the level of dysfunction of meningeal lymphatic drainage affects the severity of traumatic brain injury and the speed of recovery [105].

Finally, lactate plays an important role in the modulation of brain glucose utilization rate. Lactate uptake is highly dependent on astrocytic gap junctions. Diffusion of lactate down its concentration gradient between coupled astrocytes and their endfeet leads to discharge to perivascular fluid where it can be removed from the brain *via* perivascular flow and the GS [106, 107].

### 5.4 Immune Function

The GS, in addition to the removal of waste products and transport of substances, like the lymphatic system, is also involved in brain immunity. CNS immune surveillance is ensured by circulating T cells that can cross the BBB [108] and reach perivascular or subarachnoid spaces without neuroinflammation [109]. In the inflamed CNS tissue, they encounter tissue-resident antigen-presenting cells. Recognizing their cognate antigen on these cells leads to T cell reactivation, enabling their migration across the glial limitans into the CNS parenchyma [110]. Two-photon microscope study also showed that autoaggressive effector T cells cross the BBB, enter the subarachnoid space, and lead to neuroinflammation [111]. Meningeal lymphatic vessels could also provide a different route for trafficking immune cells and macromolecules from the central nervous system into cervical lymph nodes [107]. More recently, it was reported that circulating immune cells, such as T-cells, enter the meninges, monitor for immune signals, and drain out through the dural lymphatic vessels [112].

The pathogenesis of neuroinflammatory diseases, such as multiple sclerosis, remains unclear. The ablation of meningeal lymphatics reduced the inflammatory response of brain-reactive T-cells in an animal model of multiple sclerosis [113]. At the same time, drainage of immune cells has been reported to be important for immune surveillance of intracranial tumors. CD-8 T-cellular immunity limitation when the tumor is confined to CNS is one of the factors for uncontrolled glioblastoma growth. It was shown that ectopic expression of vascular endothelial growth factor C, a cytokine that regulates lymphangiogenesis, improves antitumor immune response [114]. In addition, disruption of meningeal lymphangiogenesis significantly reduced the efficacy of combined anti-PD-1/CTLA-4 checkpoint therapy in striatal tumor models [115]. Additional studies regarding both GS and immune brain function are needed.

Finally, astrocyte endfeet are joined together by gap junctions that allow for communication between the astrocytes. In healthy CNS, contrary to the condition of neuroinflammation, the glia limitans provide a barrier for immune cells scanning the subarachnoid and PVSs and prohibit their uncontrolled entry into the CNS parenchyma. GS permeability during inflammation and immune responses needs further research [116].

### 5.5 A Possible Route for the Spread of Cancers

There are several biological barriers that prevent systemically circulating tumor cells from seeding the CNS. They include the absence of conventional lymphatic vessels within the brain and spinal cord. The presence of the GS and its connection with intracranial lymphatic structures through the dural lymphatic vessels represent a possible route for the spread of cancers from the central nervous system. There have been several case reports describing extracranial metastasis of glioblastoma multiforme, which might be spreading *via* this route [117].

## 6 MOLECULAR TRANSPORT IN THE GLYMPHATIC SYSTEM

The GS can be seen not only as a way of the waste products and fluid outflow, but also as a delivery route. The latter is especially important for the potential use of GS to carry drugs by avoiding the BBB. PVSs are potentially filled with both CSF and ISF.

Possible routes of entry from CSF into the PVSs include stomata, which are specialized pores on the leptomeningeal cellules in the subarachnoid space of rats [12]. Intrathecally infused IgG distribution was demonstrated within perivascular space depending on the size of the antibodies in a study [118].

In the previous section, we discussed the role of AQP-4 as a determinant of the transport capacity of the GS. However, it is important to consider PVSs separated from interstitial space and CSF not only by astrocytes endfeet, but also by leptomeningeal cells. The extracellular matrix also plays an important role in the diffusion of molecules [119, 120].

As the central nervous system lacks lymphatic vessels, GS may serve as its analog for the transportation of lipophilic molecules [21]. In a healthy state, the perivascular system facilitates selective transport of small lipophilic molecules, widespread glial calcium signaling, and rapid interstitial fluid movement.

GS theory is based on the conception of neurovascular units. Experiments aimed at visualizing the PVSs involved the injection of various tracers with differing sizes and masses [121]. The findings suggested no significant size limitations for molecular transport within the PVSs. Iliff *et al*. [2] obtained important data when they studied CSF-ISF exchange. Only small molecules appeared to leave PVS to access the brain interstitium. According to one of the assumptions, selectivity was due to narrow gaps between astrocyte endfeet. At the same time, AQP-4 was shown to be the key element in CSF-ISF substances exchange (even though the AQP-4 protein only allows water to pass through) [72, 122] (Table **2**). Thus, understanding the size of molecules that could be bypassed to the CNS *via* the GS can provide insights into brain physiology and could help in creating new molecules and therapeutic options.

## 7 FLUID FLOW DIRECTION IN THE MENINGEAL LYMPHATIC VESSELS AND ITS CONNECTION WITH PERIVASCULAR SPACES

Once fluid enters the brain parenchyma, it becomes a part of the interstitial flow. The concept of interstitial flow is based on three general agreements on its physiology and includes interstitial fluid motion that mainly depends on diffusional processes in gray matter, since the major site for bulk flow in the PVSs is the white matter and that interstitial flow is an additional source of CSF [119, 123, 124]. However, the concept of bulk flow has been challenged because there is evidence that interstitial solute transport occurs by diffusion [125].

The choroid plexus produces CSF, which circulates in the subarachnoid space and is resorbed by arachnoid villi and granulations either by classical lymphatic drainage in sinonasal tissues that underlie the cribriform plate or by the meningeal lymphatics back into the systemic circulation *via* regional and cervical lymph nodes [79, 126].

Durand Fardel first theoretically described the connection between the CSF and the lymphatic system [127] in 1843.

The perivascular inflow and outflow are hypothesized to be a low-resistance route for CSF-ISF current, as the width of the PVSs is thought to be orders of magnitude greater than the interstitial space or spaces between the parenchyma of the brain [128].

The original identification of meningeal lymphatic vessels can be attributed to Paolo Mascagni, who first detailed their presence in 1783 [7]. Furthermore, it is important to acknowledge the significant contributions made by Antoine Louveau and Jonathan Kipnis, who, in October 2014, rediscovered these vessels in their study conducted at the University of Virginia, as well as the independent research conducted by Aleksanteri Aspelund, Salli Antila, and Kari Alitalo, who submitted their findings in December 2014 at the University of Helsinki [13, 64]. In a study conducted on healthy volunteers, the meningeal lymphatic vessels were visualized using MRI with gadobutrol, a gadolinium-based contrast agent. The corresponding position of the lymphatic vessels in the region of the superior sagittal and rectus sinus, and the middle meningeal arteries, was shown [129]. It was also demonstrated in the same study that morphologically meningeal lymphatics display a typical immunohistochemical panel of lymphatic endothelial markers. In a study of samples of the dura mater at autopsy, a high concentration of lymphatic vessels was revealed, and their morphology was identical to peripheral lymphatic vessels [130]. Autopsy specimens from patients with Alzheimer's disease, other types of dementia, and other neurological diseases showed that the diameter of the lymphatic vessels adjacent to the superior sagittal sinus ranged from 19 to 470 µm, and their average number exceeded 5 [131].

The confirmation of the theory about the CSF pathway to the cervical lymph nodes was shown in a study [132] that was conducted on 19 patients who were intrathecally introduced with a tracer and visualized by MRI. The maximum concentration of the tracer in the cervical nodes was noted after 24 hours in most patients. Most of the involved glymphatic activity was noted proximal to the leptomeningeal arteries. This observation confirmed, first, the role of arterial pulsation as a guiding force for the flow of glymphatic channels from the brain parenchyma to the PVSs, and second, a more significant role of meningeal lymph than nasal lymph in the drainage of CSF into the cervical lymph nodes, due to the rapid accumulation of the tracer in the superficial lymph nodes.

The direction of meningeal lymph flow was examined [133] in six healthy volunteers during MRI using an improved signal-to-noise ratio. In all patients, the meningeal lymphatic vessels were clearly visualized at the corners of the superior sagittal sinus and a competitive venous flow of meningeal lymph in the superior sagittal sinus was registered.

## 8 THE ROLE OF THE GLYMPHATIC SYSTEM IN THE DEVELOPMENT OF THE NEURODEGENERATIVE DISEASES

In view of the crucial role of the glymphatic system and its physiological complexity in brain function, data are emerging on the impact of dysfunction of the GS on neurological diseases, particularly in proteinopathies [134]. There are various mechanisms that contribute to the removal of soluble interstitial Aβ from the brain: its degradation by glial cells, neurons, or transport through the BBB and glymphatic flow of ISF through the perivascular or perineural pathways into the lymphatic channel [135] or draining veins [136]. Due to the presence of CSF dynamics in AD patients, there is a significant imbalance in the accumulation and clearance of soluble Aβ, which significantly contributes to the accumulation of Aβ in the interstitial fluid and the formation of plaques [137, 138].

With the development of this process, the accumulation of Aβ along the vessels reduces the volume of PVSs, where the glymphatic flow is formed, forming a vicious circle [139]. Post-mortem data from AD patients indicated a significant decrease in AQP-4 polarization at the endfeet of astrocytes around cerebral blood vessels, compared to relevant controls [140].

There are a significant number of studies on the dysfunction of the GS in the pathogenesis of PD, which often show oppositional results. It has been shown that an increase in PVSs is associated with worsening clinical manifestations of PD [141-143]. Various imaging modalities can be utilized to assess enlarged perivascular spaces (EPVS) and the index of diffusivity along the perivascular space (ALPS index), which may indicate the functionality of the glymphatic system and serve as potential clinical neuroimaging biomarkers for neurological disorders [144]. In addition, an increase in PVSs was associated with excessive accumulation of extracellular proteins, alpha-synuclein and tau, and dopaminergic neurodegeneration in the SN in PD. It should be noted that these studies were performed on virtually a single cohort of specific institutions. By contrast, the Donahue study [145] showed no difference in PVSs, either globally or regionally, in patients with idiopathic PD compared to healthy patients. In this case, the discordant results could be explained both by a small sample and by age and comorbid features of the comparison cohort. Alpha-synuclein accumulation negatively correlated with AQP-4 expression in the brain tissues of patients with PD, indicating a link between the development of GS dysfunction and the development of PD [146].

Cerebral edema after a stroke is another example of the important role of the GS in the pathogenesis of diseases of the central nervous system. Within minutes after an ischemic stroke, the accumulation of edema of the brain begins [147]. The GS can mediate the accumulation of edema through direct contact with astrocytes, microglia, pericytes, leukocytes, and endothelial cells in the perivascular space and the production of inflammatory cytokines. Subsequent proliferation of T-lymphocytes in the deep lymph nodes of the neck is most likely caused by the drainage of active molecules and cell fragments both through the meningeal lymphatic vessels and through the GS [148]. Further actions involve the removal of cell fragments and inflammatory products during a later stage of edema [149].

In hemorrhagic stroke, the presence of fibrin and the absence of inflow in the GS are a main factor in the development of cerebral edema. Additionally, more blood cells and clotting factors may enter the PVSs, increasing the hydrostatic pressure and osmotic gradient (Table **1**) [150].

## 9 DRUG DELIVERY *VIA* GLYMPHATIC SYSTEM

Ameliorating the delivery of drugs to the CNS has been one of the major aims of investigations for many years. Strategies to bypass the BBB could be divided into two major categories: 1) techniques that facilitate the crossing of drugs through the BBB and 2) the direct delivery to the CNS [151]. The first category includes different techniques, such as transport systems, the Trojan horse approach, nanoparticles, chimeric peptides, monoclonal antibody fusion proteins, *etc*. [152]. The delivery of drugs *via* the BBB could also be facilitated by acting on the lipophilicity and the biochemical construction of the molecule. In this regard, there are a number of monoclonal antibodies (mABs) and tyrosine kinase inhibitors (TKIs) that have demonstrated clinical activity against solid cancer metastases in the brain. Information on TKIs and mABs is provided in Tables **S1** and **S2** the second category includes intrathecal, intracerebroventricular, and intranasal routes of administration [153]. The GS could provide another possible way for the second category of the BBB bypassing strategies (either through cervical lymph nodes or as a way from the CSF to the CNS). However, while drugs could be administered intrathecally, several studies have shown that drugs do not readily pass from the CSF to the CNS parenchyma [151, 153], in contrast to what could be assumed with the GS.

The functions of MLVs and the glymphatic system are interconnected. A study utilizing MRI to evaluate the function of MLVs in humans demonstrated their association with the clearance level of the glymphatic pathway. Notably, the clearance of the glymphatic pathway was notably faster in individuals with early filling of putative MLVs compared to those with late filling [154]. It is worth noting that the functions of both MLVs and the glymphatic system decline with age.

After reviewing a large array of articles, it can be concluded that the flow of fluid is theoretically possible in the opposite direction (from the deep cervical lymph nodes to the brain through the meningeal vessels). Fluid flow in these lymphatics is due to the contraction of adjacent arterioles and the downward force of gravity [65, 155]. We assume that the change in the position of the head and neck can influence the movement of fluid (the most effective glymphatic clearance is observed in the lateral position rather than prone or supine) [16]. We propose a therapeutic route of administration involving the fluid flow from the meningeal lymphatic vessels into the CSF, then into the perivascular space, and from there into the ISF. We believe that this could be a new potential way to deliver drugs to the brain that could bypass the blood-brain barrier or in the treatment of Alzheimer's disease, stroke, and Parkinson's disease.

## 10 GLYMPHATIC SYSTEM AND BRAIN TUMORS

GS (mainly *via* the AQP-4 channel) is also proposed to play a role in the pathophysiology, clinical manifestations, and complications of brain tumors.

One of the processes that lead to the clinical findings of brain tumors is peritumoral brain edema. It is caused by primary as well as metastatic brain tumors. It leads to the elevation of ICP and the development of focal neurological deficits. Peritumoral brain edema could be explained by the higher permeability of tumor vessels and the absence of tight endothelial junctions. However, in several studies, it has also been proposed that the dysfunction of the GS (and specifically, AQP-4) could lead to the development of peritumoral brain edema [156].

The overexpression of AQP-4 channels in gliomas appears to restrict fluid flow, leading to peritumoral edema [157]. However, another study reported the level of AQP-4 in glioma to be significantly decreased. The distribution of the tracers from the subarachnoid space after cisterna magna injection to the brain parenchyma was impaired. The authors of the study proposed the para-arterial influx of subarachnoid CSF to be decreased due to the AQP-4 dysfunction in glioma [158]. This effect also led to the impediment of the intrathecal injection of the chemotherapeutic agents. Another group of researchers that used the DTI-ALPS (diffusion tensor image analysis along the perivascular space) method in 201 patients with glioma determined ALPS index (which reflects the glymphatic function) to be significantly lower in patients with higher grade glioma, larger peritumoral brain edema volumes, and IDH1 wild type gliomas. It was proposed that IDH1 wild-type glioma's more aggressive behavior leads to the disruption of interstitial fluid and consequently the remodeling of the GS, thereby leading to the lower glymphatic function of those tumors compared to the IDH1 mutant gliomas [159]. A simpler explanation of the impediment of CSF outflow in glioma was also proposed. As the tumor expands, it causes the brain tissue to occupy more intracranial space, including the subarachnoid space, which may obstruct the CSF circulation and enable the accumulation of toxic proteins, solutes, pro-inflammatory cytokines, and chemokines [160]. Thus, given the above aspects, treatment options that can restore normal CSF circulation could be potentially used in the management of brain tumors.

Another aspect of the GS dysfunction in brain tumors is the dysfunction of the meningeal lymphatic vessels. Like conventional lymphatic endothelial cells (LECs), meningeal LECs (MLECs) express the markers of CD31, VEGFR3, Prox1, PDPN, LYVE-1, and CCL21, and they efficiently drain both soluble molecules and immune cells from the subarachnoid space into CLNs [14, 113]. Some studies show that MLVs undergo extensive remodeling in mice with intracranial gliomas or metastatic melanomas [115]. On one side, they serve as a lymphatic conduit for tumor cell dissemination from CSF into CLNs. On the other side, MLVs enable DC trafficking from tumors implanted in the striatum to CLNs and CLN-mediated immune activation has been deemed essential for anti-PD-1/CTLA-4 efficacy [115].

While systemic immunotherapy has been tried in the treatment of glioblastoma multiforme, it has not been shown to be efficacious. GBM is thought to be a “cold” tumor, *i.e*., it does not induce a high level of T-cell response owing to its low immunogenicity. A group of researchers addressed this problem in an interesting way by transitioning a “cold” tumor into “hot” by using vaccines, thus introducing a higher level of antigens to the immune system and inducing a strong T-cell response specific to the tumor antigens. This approach could be used effectively to overcome the immunosuppressive environment of glioblastoma and increase efficiency [161]. GS (specifically, meningeal lymphatic vessels) was shown to connect brain tissue to the immunologically active periphery [162]. Thus, GS and vaccines could be used in conjunction to overcome the low immunogenic potential of glioblastoma (Tables **S1** and **S2**).

Given the novelty of the concept of the GS, it is not surprising that only a few potential associations between CNS tumors and this system (brain edema, possible routes of CNS tumors spreading) have been established, and that future research in this field is much needed. These associations, if further explored, may provide new therapeutic options.

## 11 THE GLYMPHATIC SYSTEM AND MENINGEAL LYMPHATICS

The glymphatic system and meningeal lymphatics are two distinct but interconnected waste clearance systems in the brain. Here are the key differences between the two:

### 11.1 Location

The glymphatic system is primarily located within the brain parenchyma (the functional tissue of the brain), whereas meningeal lymphatics are found in the meninges, the protective membranes surrounding the brain and the spinal cord [90, 107].

### 11.2 Pathway

The glymphatic system involves the flow of CSF through the brain tissue *via* perivascular spaces surrounding blood vessels. In contrast, meningeal lymphatics are lymphatic vessels within the meninges that drain interstitial fluid and waste products from the CNS into the peripheral lymphatic system [70].

### 11.3 Waste Clearance

The glymphatic system primarily removes waste products from the brain's interstitial fluid, including soluble proteins, metabolites, and byproducts of neuronal activity. It functions particularly during sleep. Meningeal lymphatics, on the other hand, primarily drain interstitial fluid and waste products from the meninges and CNS into the peripheral lymphatic system [90].

### 11.4 Implications

Dysfunction or impairment of the glymphatic system and meningeal lymphatics have been both associated with different neurological disorders. Disruptions in the glymphatic system have been linked to conditions, such as Alzheimer's disease [140] and traumatic brain injury [163], whereas abnormalities in meningeal lymphatics have been observed in multiple sclerosis [164] and other neuroinflammatory disorders [165].

Despite these differences, it is important to note that the glymphatic system and meningeal lymphatics are interconnected and work together to maintain brain health by facilitating waste clearance from the CNS. Both systems play crucial roles in removing metabolic waste and maintaining homeostasis in the brain. On the other hand, the glymphatic system and meningeal lymphatics are distinct waste clearance systems in the brain; however, there are some similarities between them, which are listed as follows:

1. Waste clearance: Both the glymphatic system and meningeal lymphatics are involved in the clearance of waste products from the central nervous system (CNS). They play crucial roles in removing metabolic waste, soluble proteins, and other harmful substances from the brain and maintaining its homeostasis [91].
2. Interstitial fluid drainage: Both systems are involved in draining interstitial fluid from the CNS. The glymphatic system clears interstitial fluid from the brain parenchyma [91], while meningeal lymphatics drain interstitial fluid from the meninges [70].
3. Connection to lymphatic system: Both the glymphatic system and meningeal lymphatics are connected to the peripheral lymphatic system. The waste products cleared by these systems eventually enter the lymphatic vessels and are transported out of the CNS.

## CONCLUSION

The GS is a relatively new concept. Its anatomic structure consists of PVSs and AQP-4 water channels. However, functions of the glymphatic system are still being vigorously explored. Recent investigations have suggested it to participate in many processes, including CSF circulation, the clearance of interstitial solutes, such as Aβ, brain immunity, intracerebral substance transport, *etc*. AQP-4 plays a pivotal role in the GS. It was observed that the loss of function of AQP-4 led to CSF tracer distribution disturbance and it was proposed to be involved in Aβ clearance impairment and brain edema development in ischemia as well as brain tumors. Impaired patency of the GS is also associated with a worse prognosis for CNS tumors and reduced effectiveness of chemotherapy [159]. This leads to the conclusion that maintaining the full functioning of the GS is necessary to maintain a healthy state of the brain.

Further study of the GS is necessary to assess its potential as a route for drug administration. In this regard, we have suggested a therapeutic route of administration involving the fluid flow from the meningeal lymphatic vessels into the CSF, then into the perivascular space, and from there into the ISF. GS is permeable to large molecules and has a much higher permeability than BBB. This capacity, combined with the fact that GS communicates with the deep neck lymph nodes, allows GS to be considered as an alternative route for drug delivery to the CNS, bypassing the BBB. More investigation is required to determine the maximum size of molecules that can be transported *via* the GS. Furthermore, the connection of the GS to the peripheral lymphatic system *via* meningeal lymphatic vessels and cervical lymph nodes is also not well understood. Therefore, to practically implement the concept of the GS and use it as a potential drug route administration, the issues highlighted need to be further studied.

## Figures and Tables

**Table 1:** Comparison of key features of the glymphatic system and meningeal lymphatic system.

**System**	**Glymphatic**	**Meningeal Lymphatics**
Primary location	Perivascular arterial and venous spaces	Alongside the venous sinuses [13, 14]
Fluid flow type	Bidirectional fluid flow [15]	Blind-ended unidirectional absorptive flow
Aquaporin-4 (AQP-4) protein	Yes	No
Fluid flow direction	CSF ↔ subarachnoid and cisternal spaces ↔ periarterial spaces ↔ aquaporin-4 (AQP-4) ↔ perivascular astrocyte end-foot processes ↔ convective flow of ISF and interstitial fluid ↔ the extracellular space ↔ perivenous spaces ↔ alongside large-caliber ventral veins ↔ subarachnoid CSF ↔ meningeal lymphatics	Subarachnoid space ↔ deep cervical lymph nodes
Fluid movement mechanism	Regulated by arterial pulsations, respiratory and sleep patterns, exercise, and position of the head [17-20]	Regulated by actively pumping collecting lymphatic vessels, extrinsic pumping, and gravity [16]
Function	Delivery of nutrients, mostly glucose, as well as delivery of circulating apolipoprotein E (from choroid plexus, astrocytic paracrine function supplies lipid molecules); clearing extracellular metabolites, such as lactate [21-23]	Clearing extracellular metabolites and waste products from CNS [15]

**Table 2:** Molecules that have been found to be transported in the glymphatic system.

**Type of Molecule**	**Model**	**Size of the Molecule**	**Distribution**	**References**
Alexa Fluor 594 hydrazide (A594)	Mice	759 Da	Quickly disseminated throughout the interstitial space	[2]
Texas Red-dextran-3 (TR-d3)	Mice	3 kDa	Entered the interstitial fluid	[2]
Fluorescein isothiocyanate-dextran-2000 (FITC-d2000)	Mice	2000 kDa	Confined to the PVSs	[2]
Alexa 647-labelled fixable dextran	Mice	10 kDa	Entered the interstitial fluid	[72]
The fluorescent dextran molecules (types D3307, D1828, D1829, and D1830). The 3-kDa dextrans were tagged with tetramethylrhodamine. The other dextrans were all tagged with Texas Red	Agarose gel and brain extracellular microenvironment	3, 10, 40, and 70 kDa	Up to 70 kDa size molecules can pass through the brain extracellular microenvironment (BEM)	[122]

## LIST OF ABBREVIATIONS

BBB: Blood-brain Barrier
CNS: Central Nervous System
CSF: Cerebrospinal Fluid
EPVS: Enlarged Perivascular Spaces
GS: Glymphatic System
ISF: Interstitial Fluid
LECs: Lymphatic Endothelial Cells
PVSs: Perivascular Spaces
SLYM: Subarachnoid Lymphatic-like Membrane
TKIs: Tyrosine Kinase Inhibitors
