# Supplementary material for: Functional and Clinical Relevance of the Crosstalk between the Glymphatic System and the Lymphatic System
Source: Curr Neuropharmacol. 2025 Jun 3;23(13):1709–25. doi: 10.2174/011570159X359861250224051857 (PMC12645143; doi:10.2174/011570159X359861250224051857)
Supplement: Supplementary file 1 [file CN-23-13-1709_SD1.pdf]

Supplementary Material

Functional and Clinical Relevance of the Crosstalk between the Glymphatic System and the Lymphatic System

Marsel Khabibov[1], Airat Garifullin[2,*], Amina Sadreeva[3], Manuel Felipe Fernandez[4], Marat Kashaev[5], Iuliia Topchu[6], Leonid Kharin[7] and Yanis Boumber[8,9,*]

[1]Department of Radiology, Dmitry Rogachev National Research Center of Pediatric Hematology, Oncology and Immunology, Moscow, 127994, Russia; [2]Department of Radiation Oncology, Republican Clinical Oncological Dispensary, Ufa, 450054, Russia; [3]I.M. Sechenov First Moscow State Medical University, Moscow, 119992, Russia; [4]Department of Medicine, Feinberg School of Medicine, Northwestern University, Chicago, IL 60611, USA; [5]Bashkir State Medical University, Ufa, 450000, Russia; [6]Robert H. Lurie Comprehensive Cancer Center, Department of Medicine, Division of Hematology/Oncology, Feinberg School of Medicine, Northwestern University, Chicago, IL 60611, USA; [7]Molecular Therapeutics Program, Fox Chase Cancer Center, Philadelphia, PA 19111, USA; [8]O'Neil Comprehensive Cancer Center, Department of Medicine, Division of Hematology/Oncology, Heersink School of Medicine, University of Alabama in Birmingham, Birmingham, AL, 35233, England; [9]Institute of Fundamental Medicine and Biology, Kazan (Volga Region) Federal University, Kazan, 420012, Russia

1875-6190/25 $65.00+.00 **© 2025 Bentham Science Publishers**

Table S1. Tyrosine kinase inhibitors that have demonstrated clinical activity against solid cancers metastases in the brain.

TKI (INN)	Target Tyrosine Kinases	Cancer Type	References
Cabozantinib	MET, VEGFR2, RET, AXL, and TYRO3	RCC	Nakagawa T, Kijima T, Imasato N, et al. Efficacy of cabozantinib therapy for brain metastases from renal cell carcinoma. IJU Case Rep. 2022;5(4):293-296. Published 2022 May 3. doi:10.1002/iju5.12459
Lapatinib	EGFR and HER2/neu receptor tyrosine kinases	Breast cancer with HER2 amplification	Lin N.U., Diéras V., Paul D., Lossignol D., Christodoulou C., Stemmler H.-J., Roché H., Liu M.C., Greil R., Ciruelos E., et al. Multicenter Phase II Study of Lapatinib in Patients with Brain Metastases from HER2-Positive Breast Cancer. Clin. Cancer Res. Off. J. Am. Assoc. Cancer Res. 2009;15:1452–1459. doi: 10.1158/1078-0432.CCR-08-1080.
Neratinib	HER2/neu receptor tyrosine kinase	Breast cancer with HER2 amplification	Stirrups R. Neratinib and Capecitabine for Breast Cancer Brain Metastases. Lancet Oncol. 2019;20:e197. doi: 10.1016/S1470-2045(19)30184-6.
Tucatinib	HER2/neu receptor tyrosine kinase	Breast cancer with HER2 amplification	Murthy R.K., Loi S., Okines A., Paplomata E., Hamilton E., Hurvitz S.A., Lin N.U., Borges V., Abramson V., Anders C., et al. Tucatinib, Trastuzumab, and Capecitabine for HER2-Positive Metastatic Breast Cancer. N. Engl. J. Med. 2020;382:597–609.
Afatinib	ErbB family of tyrosine kinases	NSCLC	Iuchi T., Shingyoji M., Sakaida T., Hatano K., Nagano O., Itakura M., Kageyama H., Yokoi S., Hasegawa Y., Kawasaki K., et al. Phase II Trial of Gefitinib Alone without Radiation Therapy for Japanese Patients with Brain Metastases from EGFR-Mutant Lung Adenocarcinoma. Lung Cancer. 2013;82:282–287.
Osimertinib	EGFR T790M mutation while sparing wild-type EGFR	NSCLC	Ramalingam S.S., Vansteenkiste J., Planchard D., Cho B.C., Gray J.E., Ohe Y., Zhou C., Reungwetwattana T., Cheng Y., Chewaskulyong B., et al. Overall Survival with Osimertinib in Untreated, EGFR-Mutated Advanced NSCLC. N. Engl. J. Med. 2020;382:41–50.
Crizotinib	anaplastic lymphoma kinase (ALK), hepatocyte growth factor receptor (HGFR, c-MET), ROS1 (c-ros), and Recepteur d'Origine Nantais (RON)	NSCLC	Shaw A.T., Bauer T.M., De Marinis F., Felip E., Goto Y., Liu G., Mazieres J., Kim D.-W., Mok T., Polli A., et al. First-Line Lorlatinib or Crizotinib in Advanced ALK-Positive Lung Cancer. N. Engl. J. Med. 2020;383:2018–2029. doi: 10.1056/NEJMoa2027187
Lorlatinib	anaplastic lymphoma kinase (ALK) as well as receptor tyrosine kinase c-ros oncogene 1 (ROS1)	NSCLC	Shaw A.T., Bauer T.M., De Marinis F., Felip E., Goto Y., Liu G., Mazieres J., Kim D.-W., Mok T., Polli A., et al. First-Line Lorlatinib or Crizotinib in Advanced ALK-Positive Lung Cancer. N. Engl. J. Med. 2020;383:2018–2029. doi: 10.1056/NEJMoa2027187
Alectinib	receptor tyrosine kinase anaplastic lymphoma kinase (ALK)	NSCLC	Novello S., Mazières J., Oh I.-J., De Castro J., Migliorino M.R., Helland Å., Dziadziuszko R., Griesinger F., Kotb A., Zeaiter A., et al. Alectinib versus Chemotherapy in Crizotinib-Pretreated Anaplastic Lymphoma Kinase (ALK)-Positive Non-Small-Cell Lung Cancer: Results from the Phase III ALUR Study. Ann. Oncol. Off. J. Eur. Soc. Med. Oncol. 2018;29:1409–1416. doi: 10.1093/annonc/mdy121
Brigatinib	ALK, ROS1, insulin-like growth factor 1 receptor and against EGFR deletions and point mutations.	NSCLC	Camidge D.R., Kim H.R., Ahn M.-J., Yang J.C.-H., Han J.-Y., Lee J.-S., Hochmair M.J., Li J.Y.-C., Chang G.-C., Lee K.H., et al. Brigatinib versus Crizotinib in ALK-Positive Non-Small-Cell Lung Cancer. N. Engl. J. Med. 2018;379:2027–2039. doi: 10.1056/NEJMoa1810171
Vemurafenib	V600E mutated BRAF protein	Melanoma	McArthur G.A., Maio M., Arance A., Nathan P., Blank C., Avril M.-F., Garbe C., Hauschild A., Schadendorf D., Hamid O., et al. Vemurafenib in Metastatic Melanoma Patients with Brain Metastases: An Open-Label, Single-Arm, Phase 2, Multicentre Study. Ann. Oncol. Off. J. Eur. Soc. Med. Oncol. 2017;28:634–641. doi: 10.1093/annonc/mdw641
Dabrafenib	BRAF V600E	Melanoma	Geukes Foppen M.H., Boogerd W., Blank C.U., Van Thienen J.V., Haanen J.B., Brandsma D. Clinical and Radiological Response of BRAF Inhibition and MEK Inhibition in Patients with Brain Metastases from BRAF-Mutated Melanoma. Melanoma Res. 2018;28:126–133. doi: 10.1097/CMR.0000000000000429.

Table S2. Monoclonal antibodies that have demonstrated clinical activity against solid cancers metastases in the brain.

M-ab (INN)	Target Protein	Brain Metastasis Activiy	References
Bevacizumab	vascular endothelial growth factor (VEGF)	NSCLC [1] , renal adenocar-cinoma [2], breast cancer [3]	1. De Braganca KC, Janjigian YY, Azzoli CG, et al. Efficacy and safety of bevacizumab in active brain metastases from non-small cell lung cancer. J Neurooncol. 2010;100(3):443-447. doi:10.1007/s11060-010-0200-2<br>2. Zustovich F, Ferro A, Lombardi G, Zagonel V, Fiduccia P, Farina P. Bevacizumab as front-line treatment of brain metastases from solid tumors: a case series. Anticancer Res. 2013 Sep;33(9):4061-5. PMID: 24023350.<br>3. Leone JP, Emblem KE, Weitz M, Gelman RS, Schneider BP, Freedman RA, Younger J, Pinho MC, Sorensen AG, Gerstner ER, Harris G, Krop IE, Morganstern D, Sohl J, Hu J, Kasparian E, Winer EP, Lin NU. Phase II trial of carboplatin and bevacizumab in patients with breast cancer brain metastases. Breast Cancer Res. 2020 Nov 30;22(1):131. doi: 10.1186/s13058-020-01372-w. PMID: 33256829; PMCID: PMC7706261.
Trastuzumab	HER2 protein	Breast cancer	Park YH, Park MJ, Ji SH, Yi SY, Lim DH, Nam DH, Lee JI, Park W, Choi DH, Huh SJ, Ahn JS, Kang WK, Park K, Im YH. Trastuzumab treatment improves brain metastasis outcomes through control and durable prolonga-tion of systemic extracranial disease in HER2-overexpressing breast cancer patients. Br J Cancer. 2009 Mar 24;100(6):894-900. doi: 10.1038/sj.bjc.6604941. Epub 2009 Feb 24. PMID: 19240719; PMCID: PMC2661774.
Rituximab	CD20 protein	CD-20 positive lymphomas	Rubenstein JL, Combs D, Rosenberg J, Levy A, McDermott M, Damon L, et al.. Rituximab therapy for CNS lymphomas: targeting the leptomeninge-al compartment. Blood. (2003) 101:466–8. 10.1182/blood-2002-06-1636
Cetuximab	epidermal growth factor receptor (EGFR)	Colorectal cancer	Fornasier G, Francescon S, Baldo P. An Update of Efficacy and Safety of Cetuximab in Metastatic Colorectal Cancer: A Narrative Review. Adv Ther. 2018;35(10):1497-1509. doi:10.1007/s12325-018-0791-0
Ipilimumab	CTLA-4 protein	Melanoma	Tawbi HA, Forsyth PA, Algazi A, Hamid O, Hodi FS, Moschos SJ, Khushalani NI, Lewis K, Lao CD, Postow MA, Atkins MB, Ernstoff MS, Reardon DA, Puzanov I, Kudchadkar RR, Thomas RP, Tarhini A, Pavlick AC, Jiang J, Avila A, Demelo S, Margolin K. Combined Nivolumab and Ipilimumab in Melanoma Metastatic to the Brain. N Engl J Med. 2018 Aug 23;379(8):722-730. doi: 10.1056/NEJMoa1805453. PMID: 30134131; PMCID: PMC8011001.
Pembroli-zumab	programmed death receptor-1 (PD-1)	NSCLC	Wakuda K, Yabe M, Kodama H, Nishioka N, Miyawaki T, Miyawaki E, Mamesaya N, Kawamura T, Kobayashi H, Omori S, Ono A, Kenmotsu H, Naito T, Murakami H, Harada H, Endo M, Gon Y, Takahashi T. Efficacy of pembrolizumab in patients with brain metastasis caused by previously untreated non-small cell lung cancer with high tumor PD-L1 expression. Lung Cancer. 2021 Jan;151:60-68. doi: 10.1016/j.lungcan.2020.11.009. Epub 2020 Nov 15. PMID: 33246646.
Nivolumab	PD-1 protein	NSCLC	Crinò L, Bronte G, Bidoli P, Cravero P, Minenza E, Cortesi E, Garassino MC, Proto C, Cappuzzo F, Grossi F, Tonini G, Sarobba MG, Pinotti G, Numico G, Samaritani R, Ciuffreda L, Frassoldati A, Bregni M, Santo A, Piantedosi F, Illiano A, De Marinis F, Tamberi S, Giannarelli D, Delmonte A. Nivolumab and brain metastases in patients with advanced non-squamous non-small cell lung cancer. Lung Cancer. 2019 Mar;129:35-40. doi: 10.1016/j.lungcan.2018.12.025. Epub 2019 Jan 15. PMID: 30797489.
